# From microbiome signatures to precision oncology in colorectal cancer

**DOI:** 10.3389/fonc.2026.1926171

**Published:** 2026-09-24

**Authors:** Lodovica Langellotti, Carlo Alberto Schena, Marcello Covino, Sergio Alfieri, Fausto Rosa

**Affiliations:** 1 Department of Digestive Surgery, Fondazione Policlinico Universitario Gemelli IRCCS, Rome, Italy; 2 Department of General Surgery, Fondazione Poliambulanza Istituto Ospedaliero, Brescia, Italy; 3 Department of Translational Medicine and Surgery, Facoltà di Medicina e Chirurgia, Università Cattolica del Sacro Cuore, Rome, Italy

**Keywords:** colorectal cancer, engineering, microbiome, oncology, precision oncology

## 1 The microbiome comes of age

Over the past decade, the gut microbiome has evolved from a promising area of investigation to one of the most compelling biological dimensions of colorectal cancer (CRC) (1, 2). Numerous studies have consistently identified microbial dysbiosis in patients with CRC, with recurrent enrichment of oral-associated species such as *Fusobacterium nucleatum*, *Parvimonas micra*, and *Peptostreptococcus stomatis* (3–5). These observations have fueled growing interest in the microbiome as a source of non-invasive biomarkers and a potential therapeutic target. Yet one fundamental question has persisted: are these microbial signatures sufficiently robust and reproducible to support clinical translation?

The large international metagenomic analysis by Piccinno and colleagues (6) provides one of the strongest answers to date. By integrating 3,741 stool metagenomes from 18 independent cohorts across 11 countries, the authors demonstrate that microbiome-based classification of CRC is remarkably reproducible across diverse populations while simultaneously revealing biologically meaningful heterogeneity. Beyond confirming previously recognized CRC-associated taxa, the study incorporates stage-specific analysis, tumor location, and strain-level resolution, highlighting the complexity of host–microbe interactions and the importance of moving beyond species-level descriptions.

The significance of this work extends beyond the identification of additional microbial biomarkers. Rather, it marks an important turning point for the field. For years, CRC microbiome research has been challenged by concerns regarding cohort-specific effects, methodological variability, and inconsistent reproducibility. By demonstrating that a core microbial signal persists across thousands of individuals, Piccinno and colleagues largely settle the debate over whether a reproducible CRC-associated microbiome exists. The question is therefore no longer whether a CRC-associated microbiome exists, but how these microbial signatures can be translated into mechanistic insights capable of informing precision oncology. This transition—from microbial discovery to microbial intervention—is likely to define the next phase of cancer microbiome research, in which microbial communities evolve from disease biomarkers into biological targets and potentially programmable therapeutic tools. The conceptual evolution discussed throughout this article is summarized in **Figure 1**.

**Figure 1:**
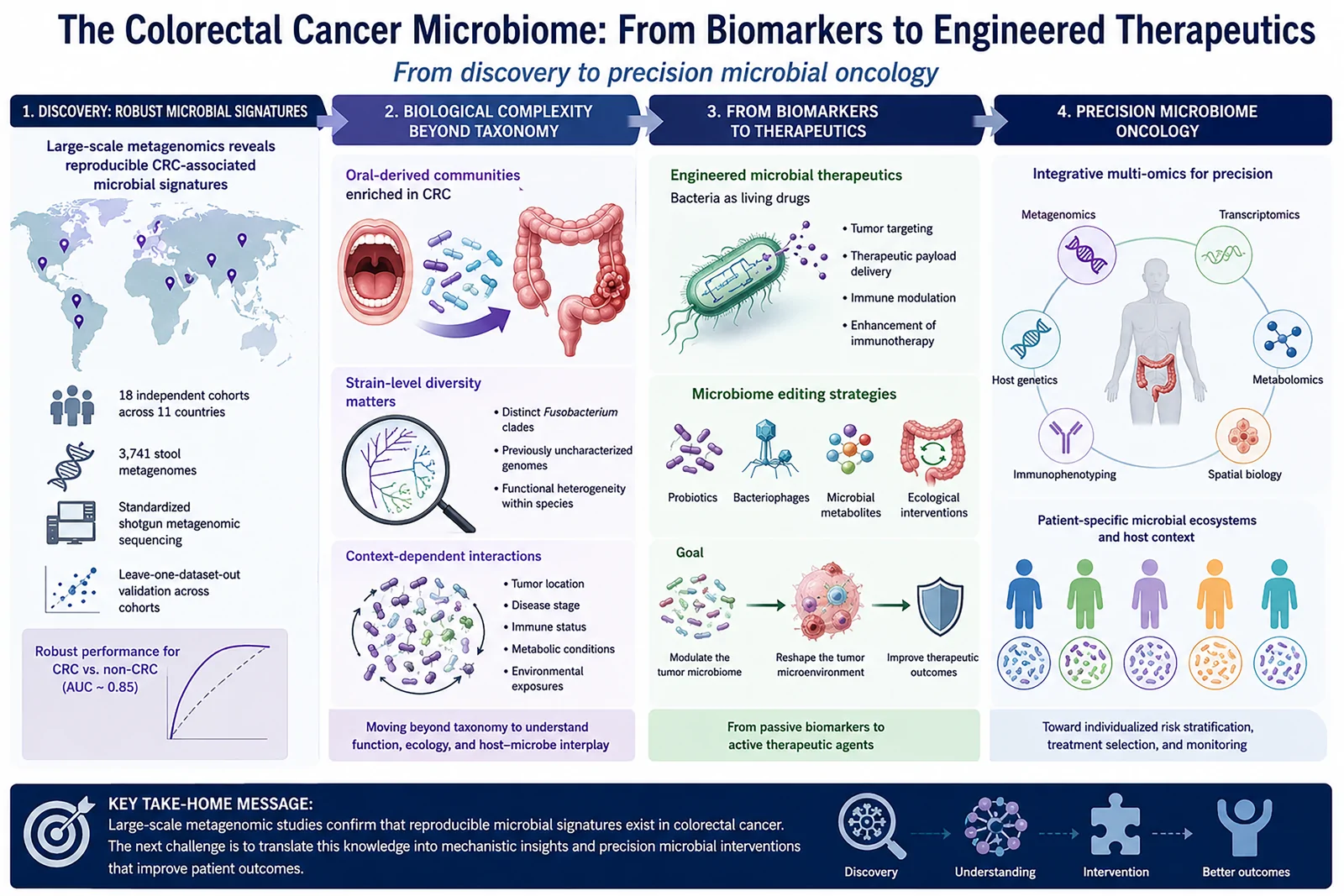
Conceptual framework illustrating the evolution of colorectal cancer microbiome research from reproducible microbial biomarkers to engineered microbial therapeutics and precision microbial oncology.

## 2 Beyond microbial signatures: embracing biological complexity

One of the most important contributions of the study by Piccinno and colleagues (6) is not simply the confirmation of previously described CRC-associated microorganisms, but the demonstration that biologically relevant information lies beyond conventional taxonomic profiling. While *Fusobacterium nucleatum* has become the emblematic microbial biomarker of colorectal cancer (4, 5, 7), the present work highlights a more complex microbial ecosystem characterized by oral-derived communities, strain-level diversity, and context-dependent microbial interactions (6). This shift challenges the long-standing tendency to interpret individual bacterial species as isolated biomarkers and instead encourages a more ecological view of the tumor microbiome.

The incorporation of strain-resolved metagenomics represents a particularly significant advance (6). Bacterial species encompass substantial genomic and functional heterogeneity, with individual strains differing in virulence factors, metabolic capabilities, immune-modulating properties, and colonization potential (7, 8). Consequently, assigning biological significance at the species level may obscure clinically relevant variation. The identification of distinct *Fusobacterium* clades and previously uncharacterized microbial genomes associated with colorectal cancer suggests that microbial functionality, rather than taxonomy alone, is likely to determine disease-associated phenotypes (6).

Equally important is the recognition that microbial signatures should be interpreted within the context of the host. Tumor location, disease stage, immune status, metabolic conditions, and environmental exposures all contribute to shaping the intestinal ecosystem and may influence both microbial composition and function (6, 9). Rather than merely representing biological variability, this heterogeneity may explain why similar microbial taxa display different clinical associations across individual patients. Future investigations should therefore move beyond cataloguing microorganisms toward integrating microbial genomic features with host molecular characteristics, immune landscapes, and tumor biology (9, 10).

This systems-level perspective is particularly relevant for translational oncology. Understanding not only which microorganisms are present, but also how they interact with the host and with one another, will be essential for developing reliable biomarkers and identifying microbial targets suitable for therapeutic manipulation (8–10). As the field progresses, functional microbiomics, multi-omics integration, and computational modeling are likely to become indispensable tools for transforming descriptive microbial signatures into clinically actionable biological knowledge (6, 9, 10).

## 3 From microbial biomarkers to engineered microbial therapeutics

The transition from microbiome discovery to clinical implementation represents one of the most exciting challenges in contemporary oncology. While the identification of reproducible microbial biomarkers is an essential first step, the goal extends far beyond diagnosis. A detailed understanding of microbial ecology, functional genomics, and host–microbe interactions are expected to provide the biological foundation for the development of innovative therapeutic strategies capable of modulating the tumor microenvironment (5–10).

In this context, studies such as that of Piccinno and colleagues have implications that reach well beyond colorectal cancer screening. By identifying reproducible microbial communities, strain-specific associations, and ecological patterns linked to disease progression, they generate the mechanistic knowledge required to move toward rational microbiome manipulation (6). Rather than considering microorganisms as passive biomarkers, these findings support a paradigm in which microbes become active therapeutic targets—or even therapeutic agents themselves (9–11).

Recent advances in synthetic biology and microbial engineering are accelerating this transition. Engineered bacterial strains are being developed to selectively colonize tumors, deliver therapeutic payloads, modulate local immune responses, and enhance the efficacy of immune checkpoint inhibitors while minimizing systemic toxicity (11). At the same time, microbiome editing through probiotics, bacteriophages, microbial metabolites, or targeted ecological interventions is emerging as a promising strategy to reshape tumor-associated microbial communities. These approaches share a common prerequisite: a comprehensive understanding of which microorganisms are present, which functions they perform, and how they interact with both the host immune system and the tumor ecosystem (5–7, 9, 10).

The future of precision oncology will therefore depend not only on identifying reproducible microbial signatures, but also on translating these biological insights into targeted interventions. Integrating metagenomics with transcriptomics, metabolomics, spatial biology, and immunophenotyping will be essential to identify microbial functions that can be therapeutically exploited (2, 5–7). In this perspective, the microbiome evolves from a diagnostic biomarker into a dynamic and potentially programmable component of cancer therapy. The challenge ahead is no longer simply to describe the tumor-associated microbiome, but to understand how it can be manipulated safely and effectively to improve patient outcomes (11, 12).

## 4 The next frontier: from precision microbiomics to precision microbial oncology

The remarkable progress achieved in CRC microbiome research over the past decade suggests that the field is approaching a pivotal transition. Large-scale metagenomic studies have established that reproducible microbial signatures exist across diverse populations, while advances in strain-level profiling and multi-omics technologies are beginning to uncover the functional complexity of tumor-associated microbial ecosystems (4–7). The next challenge is to translate these discoveries into clinically meaningful interventions (6).

Achieving this goal will require a shift from descriptive microbiome research toward functional and mechanistic investigations. Future studies should integrate microbial genomics with host genetics, immune profiling, metabolomics, and spatial transcriptomics to identify the molecular pathways through which microbial communities influence tumor initiation, progression, and therapeutic response (5–7, 12). Such integrative approaches will provide the biological framework necessary to identify patients most likely to benefit from microbiome-informed strategies and to define biomarkers with genuine predictive value (5, 6, 12).

Equally important will be the development of personalized approaches that acknowledge the substantial interindividual variability of the human microbiome. Rather than searching for universal microbial signatures, future research should embrace patient-specific microbial ecosystems and their dynamic interactions with the host (6, 12). Precision microbiomics may therefore become an essential component of precision oncology, enabling individualized risk stratification, treatment selection, and therapeutic monitoring (12).

Ultimately, the greatest impact of microbiome research may not arise from identifying which microorganisms are associated with cancer, but from understanding how microbial communities can be rationally manipulated to improve clinical outcomes. Engineered bacteria, live biotherapeutic products, microbiome editing technologies, and combination strategies with immunotherapy represent promising avenues that are rapidly moving from experimental concepts toward clinical reality (11). In this evolving landscape, studies such as that of Piccinno and colleagues provide more than robust biomarkers—they establish the biological foundation upon which the next generation of precision microbial oncology can be built (6, 12).
